# Draft Genome Sequence of *Lactobacillus crispatus* EM-LC1, an Isolate with Antimicrobial Activity Cultured from an Elderly Subject

**DOI:** 10.1128/genomeA.01070-13

**Published:** 2013-12-19

**Authors:** Susan E. Power, Hugh M. B. Harris, Francesca Bottacini, R. Paul Ross, Paul W. O’Toole, Gerald F. Fitzgerald

**Affiliations:** School of Microbiology, University College Cork, Cork, Irelanda; Alimentary Pharmabiotic Centre, University College Cork, Cork, Irelandb; Teagasc Food Research Centre, Moorepark, Fermoy, County Cork, Irelandc

## Abstract

Here we report the 1.86-Mb draft genome sequence of *Lactobacillus crispatus* EM-LC1, a fecal isolate with antimicrobial activity. This genome sequence is expected to provide insights into the antimicrobial activity of *L. crispatus* and improve our knowledge of its potential probiotic traits.

## GENOME ANNOUNCEMENT

Lactobacilli are subdominant members of the human gut microbiota and encompass a considerable number of different species that display a relatively high degree of diversity (1). A number of *Lactobacillus* species are considered to have probiotic properties, offering beneficial roles in maintaining the health status of the host (2). *Lactobacillus crispatus* can persist in the gastrointestinal tract (3) and is among the most prevalent species of the *Lactobacillus*-dominated human vaginal microbiota (4). *L. crispatus* EM-LC1 was isolated from a fecal sample obtained from an elderly subject and showed H_2_O_2_-independent antimicrobial activity in cell-free supernatant. The genome sequence of this strain will provide a genomic platform for investigation of *L. crispatus* antimicrobial activity and will elucidate the genetic basis for its potential probiotic traits.

The genome was sequenced at Macrogen (Seoul, South Korea) on the Illumina platform, generating a paired-end library containing 35,397,530 reads of 101 bp. The data were assembled into 54 scaffolds using the *de novo* assembly program Velvet (5). MAUVE was used to reorder scaffolds based on the reference genome of *L. crispatus* ST1 (6). tRNAs were identified using tRNA-scan SE (7). Protein coding regions were predicted using Metagene (8), and annotation was subsequently performed on the basis of BLASTP (9) analysis against a nonredundant protein database (nr) provided by the National Center for Biotechnology Information (NCBI). This automated annotation was then manually curated in Artemis (10). A functional classification was applied using the Clusters of Orthologous Groups (COG) database (11). rRNA operons were detected on the basis of BLASTN searches and annotated manually.

The draft genome sequence of *L. crispatus* EM-LC1 consists of 1,862,161 bp, with a G+C content of 37%, which is similar to those of other *Lactobacillus* genomes (6, 12). All predicted genes, proteins, enzymes, and their functions are putative. The draft genome sequence contains 1,827 protein-encoding genes, representing a coding density of 87.6%, with an average gene length of 893 bp. No rRNA locus was assembled due to exclusion of repetitive sequences from the assembly. Forty-five tRNA genes and 27 transposase-encoding genes were identified. No complete prophages were found in the genome sequence, but a number of single open reading frames (ORFs) with similarity to phage genes were identified. Genes encoding a helveticin-like bacteriocin and a class II bacteriocin-like product were also identified.

Functional classification of the predicted genes by COG (11) showed that 1,383 predicted protein-encoding genes (75.7%) were homologous to members of known gene families, including 155 (8.5%) identified as “general function prediction only” and 130 (7.1%) with poorly characterized gene functions designated “function unknown.”

### Nucleotide sequence accession numbers.

This draft whole-genome shotgun project has been deposited in GenBank under the accession no. AXLM00000000. The version described in this paper is the first version, AXLM01000000.
